# Ist die Polytraumaversorgung im aG-DRG-System defizitär?

**DOI:** 10.1007/s00113-021-01015-5

**Published:** 2021-06-08

**Authors:** Nikolas Schopow, Anja Botzon, Kristian Schneider, Carolin Fuchs, Christoph Josten, Nikolaus von Dercks, Johannes Fakler, Georg Osterhoff

**Affiliations:** 1 Klinik für Orthopädie, Unfallchirurgie und Plastische Chirurgie, Universitätsklinikum Leipzig, Liebigstraße 20, 04103 Leipzig, Deutschland; 2 Bereich 3 – Finanzen, Planung und Controlling, Universitätsklinikum Leipzig, Liebigstraße 18, 04103 Leipzig, Deutschland; 3 Klinik für Allgemein Orthopädie und Tumororthopädie, Universitätsklinikum Münster, Albert Schweitzer Campus 1, 48149 Münster, Deutschland

**Keywords:** Schockraum, Schwerstverletzt, Ökonomische Evaluation, Finanzielle Analyse, Behandlungskosten, Shock room, Severely injured, Economic evaluation, Financial analysis, Treatment costs

## Abstract

**Hintergrund:**

Die interdisziplinäre Versorgung von polytraumatisierten PatientInnen ist personal- und ressourcenaufwendig. Seit der Einführung des G‑DRG-Systems 2003 in Deutschland wurde in den meisten Untersuchungen ein finanzielles Defizit in der Schwerstverletztenversorgung festgestellt. Ziel dieser Studie war es, Auswirkungen des 2020 neu eingeführten aG-DRG-Systems auf die Kostendeckung in der Schwerverletztenbehandlung zu analysieren. Erstmals wurden auch die Kosten für Organisation, Zertifizierung und Dokumentation sowie die Kosten für nicht schwer verletzte SchockraumpatientInnen betrachtet.

**Methodik:**

Eingeschlossen wurden alle PatientInnen, die im Jahr 2017 im chirurgischen Schockraum der Zentralen Notaufnahme des Universitätsklinikums Leipzig behandelt wurden. Für die Analyse wurden das Kostenmodell nach Pape et al. um die Module Organisation, Dokumentation und Zertifizierung ergänzt sowie die Kosten für „übertriagierte“ PatientInnen betrachtet. Es erfolgte die Berechnung der Kosten in den Jahren 2017–2020 im Vergleich der jeweiligen Erlöse.

**Ergebnisse:**

Es wurden 834 PatientInnen im Schockraum behandelt. Die 258 schwer verletzten PatientInnen wurden in 3 Gruppen untergliedert: „ISS 9–15 + ITS“ (*n* 72; ∅ ISS 11,9; Kosten/PatientIn 14.715 €), „ISS ≥ 16“ (*n* 186; ∅ ISS 27,7; Kosten/PatientIn 30.718 €) und „DRG-Polytrauma“ (*n* 59; ∅ ISS 32,4; Kosten/PatientIn 26.102 €).

**Schlussfolgerung:**

Die Polytraumaversorgung im aG-DRG 2020 ist defizitär. Insgesamt entstand im Jahr 2020 ein Defizit von 5858 € pro schwer verletztem/verletzter PatientIn.

**Zusatzmaterial online:**

Die Online-Version dieses Beitrags (10.1007/s00113-021-01015-5) enthält zusätzliche Tabellen zur Kostenberechnung sowie detaillierte Informationen zur Methode, weitere Ergebnisse und weiterführende Literatur. Beitrag und Zusatzmaterial stehen Ihnen auf www.springermedizin.de zur Verfügung. Bitte geben Sie dort den Beitragstitel in die Suche ein, das Zusatzmaterial finden Sie beim Beitrag unter „Ergänzende Inhalte“.

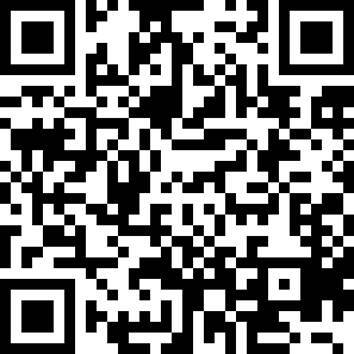

## Abstract (en)

**Background:**

The interdisciplinary care of severely injured patients is staff and resource intensive. Since the introduction of the G‑DRG system in Germany in 2003, most studies have identified a financial deficit in the care of severely injured patients. The aim of this study was to analyze the effects of the new aG-DRG system introduced in 2020 on cost recovery in the treatment of severely injured patients. For the first time, the costs for organization, certification and documentation as well as the costs for non-seriously injured shock room patients were included.

**Methods:**

All patients who were treated in the surgical shock room of the emergency department of the Leipzig University Hospital in 2017 were included. For the analysis, the cost model according to Pape et al. was extended by the module organization, documentation and certification and for the first time the costs for overtriaged patients were considered. A cost calculation was performed for the years 2017–2020 as well a comparison with the respective earnings.

**Results:**

A total of 834 patients were treated in the shock room and 258 severely injured patients were divided into 3 groups: ISS 9–15 + ICU (*n* 72; ∅ ISS 11.9; costs per patient 14,715 €),ISS ≥ 16 (*n* 186; ∅ ISS 27.7; costs per patient 30,718 €) and DRG polytrauma (*n* 59; ∅ ISS 32.4; costs per patient 26,102 €).

**Conclusion:**

Polytrauma care under the aG-DRG 2020 is in deficit. Overall, in 2020 a deficit of 5858 € per severely injured patient resulted.

## Einleitung

Eine Kernkompetenz eines überregionalen Traumazentrums (ÜTZ) ist die Versorgung von schwer verletzten und polytraumatisierten PatientInnen. Die Versorgung Schwerverletzter setzt eine interdisziplinäre Zusammenarbeit voraus und ist mit hohen Personal‑, Material- und Vorhaltekosten verbunden. Notaufnahme, OP und Intensivstation eines ÜTZ müssen rund um die Uhr bereit sein, 2 Schwerverletzte zeitgleich bestmöglich zu versorgen, dafür sollte beispielsweise das Schockraumteam folgende Personen beinhalten: Basisteam (sofort anwesend): 2*UnfallchirurgInnen, 1*AnästhesistIn, 3*Pflegekräfte, 1*Medizin-technische Radiologieassistenz (MTRA); erweitertes Team (zusätzlich innerhalb von 30 min): OberärztInnen für spezielle Unfallchirurgie, Anästhesie, Neurochirurgie und Radiologie sowie 2*OP-Pflegekräfte; Spezialisten aus 12 weiteren Fachdisziplinen sollten vor Ort vorgehalten werden [24].

Diese Ressourcen werden für die Versorgung von Schwerverletzten bereitgehalten, dabei stehen Traumazentren vor der Herausforderung, Fälle unterschiedlicher Behandlungs- und Kostenintensität miteinander zu vereinbaren. Durch die Umstellung auf das German-Diagnosis-Related-Groups(G-DRG)-System in Deutschland im Jahr 2003 wurde ein Fallkostenpauschalsystem für die Abrechnung der stationären PatientInnen eingeführt. Dies führte dazu, dass unterschiedliche Behandlungsfälle bei der Abrechnung mit den Krankenkassen in Gruppen gleicher Diagnosen zusammengefasst werden. Mit Einführung der aG-DRG im Jahr 2020 werden die Pflegepersonalkosten in den DRG-Fallpauschalen ausgegliedert und über ein krankenhausindividuelles Pflegebudget nach dem Selbstkostendeckungsprinzip finanziert.

In der klinischen Versorgung wurde ein Polytrauma ursprünglich definiert als das gleichzeitige Vorhandensein von Verletzungen verschiedener Körperregionen, von denen mindestens eine Verletzung oder aber die Kombination mehrerer Verletzungen als lebensbedrohlich einzuschätzen ist [1]. Wissenschaftlich existieren weitere Definitionen wie ein Injury Severity Score (ISS) ≥ 16 oder die Berlin-Definition [2].

Im G‑DRG-System wird die Diagnose Polytrauma zusammengefasst unter der „major diagnostic category“ (MDC) 21A und in 3 Basis-DRG unterteilt (Zusatzmaterial online: Tab. 1) [3–7].

Durch die Unterschiede in den Definitionen kommt es zu einer „Fehlgruppierung“ von bis zu 30 % der polytraumatisierten Patienten in andere Hauptdiagnosen [8].

Bereits vor der Einführung der Fallkostenpauschalen wurden die ökonomischen Aspekte der Schwerverletztenversorgung national und international diskutiert [9, 10]. Qvick et al. zeigten, dass die Umstellung von der tagessatzbasierten Abrechnung auf die fallkostenbasierte Abrechnung einer um 34 % verringerten Vergütung der jeweiligen Versorgungsfälle gleichkommt [11].

In den bisher zu diesem Thema publizierten Studien wurden Verluste von −2565 bis zu −12.893 € je Fall aufgezeigt [12–17]. Dabei wurde zur Berechnung der tatsächlichen Behandlungskosten polytraumatisierter PatientInnen entweder eine Einzelkostenabrechnung entsprechend den Vorgaben des Instituts für das Entgeltsystem im Krankenhaus (InEK) durchgeführt, oder die Kosten wurden auf Behandlungszeiten in den einzelnen Zwischenschritten der Therapie heruntergerechnet. In keiner der bisherigen Veröffentlichungen wurden allerdings die Kosten der Organisation von Verlegungen externer PolytraumapatientInnen, die Zusatzkosten durch „Übertriagierung“ und der Mehraufwand durch Dokumentationspflichten und Zertifizierungskosten hinreichend berücksichtigt.

Ziel dieser Arbeit ist daher eine Kosten-Erlös-Analyse der Behandlung Schwerverletzter, basierend auf einem nachvollziehbaren, detaillierteren und umfassenderen Modell. Für Krankenhäuser ohne Einzelkostenabrechnung ist eine modellbasierte Berechnung eine sinnvolle und umsetzbare Lösung zur Erfassung der Behandlungskosten. Vergleichend erfolgt die Berechnung entsprechend dem Kostenschätzer des TraumaRegister DGU® [12].

## Methode

### Patientenkollektiv und Auswahlkriterien

Eingeschlossen wurden alle PatientInnen, die im Jahr 2017 im chirurgischen Schockraum der Zentralen Notaufnahme des Universitätsklinikums Leipzig (UKL) behandelt wurden. Eine detaillierte Kostenanalyse erfolgte an allen primär behandelten oder sekundär zuverlegten PatientInnen, die im TraumaRegister DGU® dokumentiert wurden. Im TraumaRegister DGU® wurden diejenigen Patienten dokumentiert, die nach Behandlung im Schockraum in einen OP oder auf die Intensivstation verlegt wurden und darüber hinaus mindestens einen ISS ≥ 9 aufwiesen.

### Berechnung der Kosten

**Figure 1:**
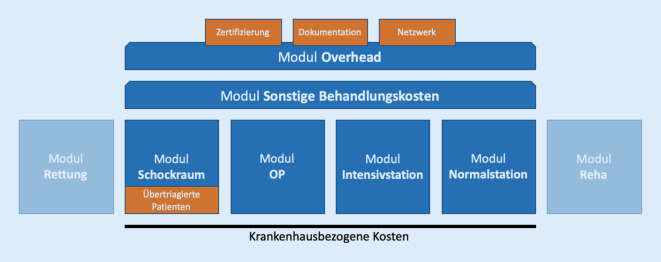

Die Gesamtkosten der Schwerverletztenbehandlung setzen sich aus mehreren Kostenfaktoren zusammen. Für die hier vorliegende Berechnung wurde das Berechnungsmodell nach Pape et al. um die Kosten für übertriagierte Schockraumpatienten, Zertifizierungs‑, Dokumentations- und Netzwerkkosten ergänzt (Abb. 1; [18]). Alle dokumentierten Behandlungen, Aufenthaltszeiten, Materialien und Diagnosemittel wurden aus dem Krankenhausinformationssystem, der Auswertung des TraumaRegister DGU® der PatientInnen des UKL sowie der klinikinternen Schockraumdokumentation erfasst und ausgewertet. Die detaillierte Berechnung der Module („Schockraum“, „OP“, „Intensivstation“, „Normalstation“, „Sonstige Behandlungskosten“ und „Overhead“ finden Sie im Zusatzmaterial online: ESM10: Detaillierte Methode, weitere Ergebnisse, weiterführende Literatur).

### Berechnung der Erlöse

Die eingeschlossenen Fälle wurden in die DRG-Systeme 2017–2020 eingruppiert und die jeweiligen Relativgewichte berechnet. Die jeweiligen Zusatzentgelte wurden den Krankenhausfällen aufaddiert. Die Erlöse wurden anhand der Landesbasisfallwerte Sachsens (LBFW) von 2017–2020 berechnet (Zusatzmaterial online: Tab. 6). Die Diagnose DRG B61B (bestimmte akute Erkrankungen und Verletzungen des Rückenmarks ohne komplexen Eingriff oder mehr als 13 Belegungstage oder nicht wegverlegt) ist nicht in dem Fallpauschalenkatalog vergüteter, vollstationärer Leistungen enthalten. Es handelt sich um ein krankenhausindividuelles Entgelt nach § 6 Abs. 1 Satz 1 des Krankenhausentgeltgesetzes. Für diese Diagnose wurde der am Universitätsklinikum Leipzig vereinbarte Betrag als Rechnungsgrundlage in Höhe von 12.157,93 € angenommen.

Die fiktiven Erlöse für die Jahre 2018–2020 wurden mithilfe von Übergangsgroupern berechnet (Fa. ID Berlin, Berlin, Deutschland).

Die Berechnungsmethode und Kalkulationsdaten des Kostenschätzers im TraumaRegister DGU® finden Sie im Zusatzmaterial online: ESM10: Detaillierte Methode, weitere Ergebnisse, weiterführende Literatur [12].

#### Statistische Analysen

Kontinuierliche Daten wurden als Mittelwert mit Standardabweichung dargestellt, diskrete Daten als absolute und prozentuale Zahl. Die Berechnung der Mittelwerte, Standardabweichung und Signifikanztestung (*t*-Test und Chi-Quadrat-Test) erfolgten in SPSS (Fa. IBM, Armonk, NY, USA). Als statistisch signifikant wurden *p*-Werte < 0,05 gewertet.

## Ergebnisse

### Patientenkollektiv

**Figure 2:**
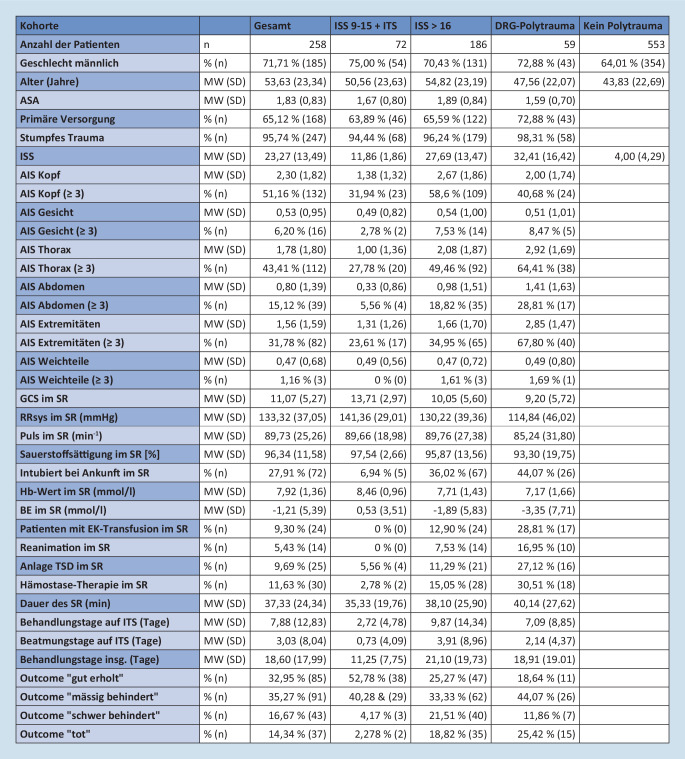

**Figure 3:**
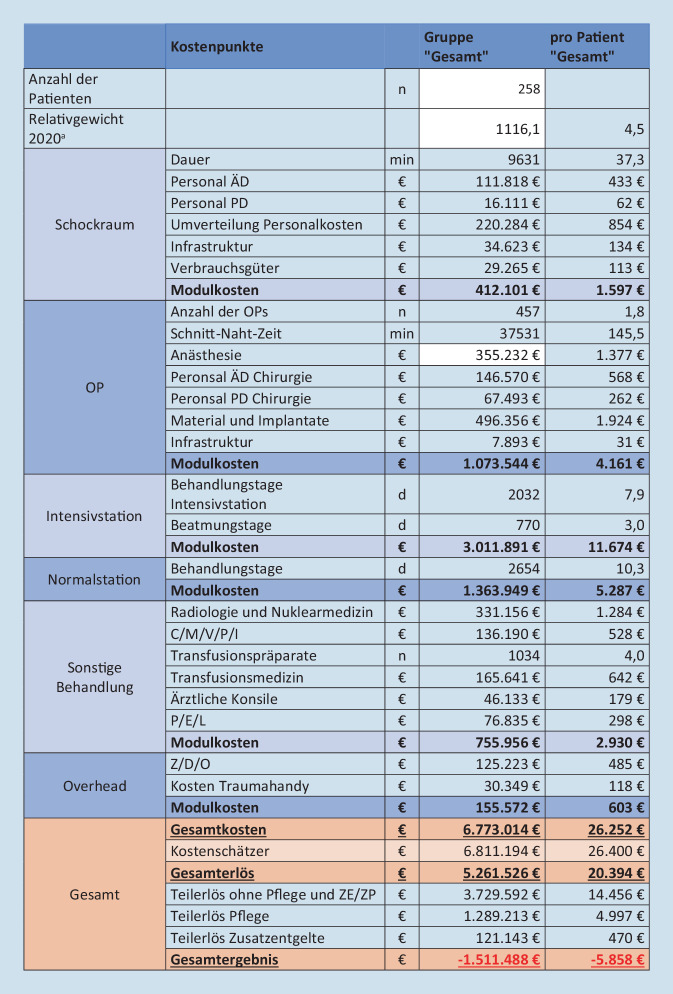

Im Beobachtungszeitraum wurden 834 PatientInnen primär oder sekundär über den Schockraum aufgenommen (Abb. 2). Davon wurden 258 PatientInnen als schwer verletzt eingestuft, im TraumaRegister DGU® (ISS 9–15 + ITS oder ISS ≥ 16) entsprechend dokumentiert und in der vorliegenden Arbeit in der Gruppe „Gesamt“ zusammengefasst. Diese PatientInnen wurden für die Analyse in 2 Kohorten weiter aufgeteilt: nichtpolytraumatisierte Schwerverletzte „ISS 9–15 + ITS“ (*n* 72; ∅ ISS 11,9 ± 1,8; ISS_min_ 9–ISS_max_ 15) und polytraumatisierte Patienten „ISS ≥ 16“ (*n* 186; ∅ ISS 27,7 ± 13,5; ISS_min_ 16–ISS_max_ 75). Zwischen diesen beiden Gruppen gab es keinen signifikanten Unterschied bei Alter (*p*_ISS_ 0,188), Geschlecht (*p*_ISS_ 0,539) und Vorerkrankungen (ASA-Score) (*p*_ISS_ 0,136). Zusätzlich wurden zur DRG-bezogenen Betrachtung alle PatientInnen mit der Hauptdiagnose MCD 21A gruppiert in der Gruppe „DRG-Polytrauma“ (*n* 59; ∅ ISS 32,4 ± 16,4; ISS_min_ 13–ISS_max_ 75). Im Vergleich zwischen der Gruppe „DRG-Polytrauma“ und den restlichen PatientInnen aus der Gruppe „Gesamt“ besteht ein signifikanter Unterschied im Altersdurchschnitt (*p*_DRG_ 0,035). Kein signifikanter Unterschied besteht bei der Geschlechterverteilung (*p*_DRG_ 0,749) und zwischen den ASA-Scores (*p*_DRG_ 0,103). In der Gruppe „kein Polytrauma“ sind die PatientInnen enthalten, die zwar über den chirurgischen Schockraum, aber ohne entsprechendes Verletzungsmuster angenommen wurden (*n* 553; ∅ ISS 4,0 ± 4,3; ISS_min_ 0–ISS_max_ 15). Wenn nicht anders angegeben, beziehen sich die im weiteren Fließtext angegebenen Werte auf das Jahr 2020. Dargestellt werden jeweils die Gesamtkosten/-erlöse (Kosten/Erlöse pro PatientIn) der Gruppe „Gesamt“ (Abb. 3), die einzelnen Kostenpunkte und Subgruppen des Patientenkollektivs sind im Zusatzmaterial online: Tab. 7 aufgeschlüsselt.

#### Modul „Schockraum“

Trotz der variierenden Behandlungsintensität bestand klinisch und statistisch kein signifikanter Unterschied in der durchschnittlichen Dauer der Schockraumbehandlung zwischen den Gruppen (35–40 min, *p*_ISS_ 0,411 und *p*_DRG_ 0,365). Aus der Gruppe „ISS 9–15 + ITS“ war kein/keine PatientIn bei der Übergabe im Schockraum reanimationspflichtig, und nur in 6 % der Fälle wurde eine Thoraxsaugdrainage angelegt. Demgegenüber waren 17 % der PatientInnen der Gruppe „DRG-Polytrauma“ reanimationspflichtig, und 27 % benötigten mindestens eine Thoraxsaugdrainage. In der Schockraumphase erhielten keiner/keine der PatientInnen der Gruppe „ISS 9–15 + ITS“, aber 13 % der Gruppe „ISS ≥ 16“ und 29 % der Gruppe „DRG-Polytrauma“ eine Bluttransfusion.

Für die Behandlung im Schockraum entstanden Kosten von 412.101 € (1597 €/PatientIn) (Abb. 3). Dabei enthalten sind 220.284 € (845 €) für den überschüssigen Personaleinsatz bei „übertriagierten“ PatientInnen. Dies entspricht einem Anteil von 53,4 % der gesamten Modulkosten bzw. 3,3 % der Gesamtkosten.

#### Modul „OP“

Ein signifikanter Unterschied innerhalb der Subgruppen zeigte sich bei der Anzahl der durchgeführten Operationen. PatientInnen der Gruppe „ISS 9–15 + ITS“ wurden im Durchschnitt 1,2-mal operiert, gegenüber 2,0 Operationen in der Gruppe „ISS ≥ 16“ (*p*_ISS_ 0,038) und 2,6 Operationen in der Gruppe „DRG-Polytrauma“ (*p*_DRG_ 0,004). Die Dauer pro Operation lag bei 81–86 min pro Eingriff in den Subgruppen. Insgesamt entstanden Operationskosten von 1073.544 € (4161 €/PatientIn).

#### Modul „Intensivstation“

Zwischen den Gruppen „ISS9–15 + ITS“ und „ISS ≥ 16“ bestehen signifikante Unterschiede in der Behandlungsdauer (2,72–9,87 Tage, *p*_ISS_ < 0,001, *p*_DRG_ 0,672) und der invasiven Beatmungsdauer (0,71–3,97 Tage, *p*_ISS_ 0,004, p_DRG_ 0,345) der PatientInnen. Es entstanden Kosten für eine intensivmedizinische Betreuung in Höhe von 3011.891 € (11.674 €/PatientIn).

#### „Normalstation“

Die PatientInnen wurden im Durchschnitt 10,3 Tage (9,2–11,4 Tage) auf Normalstationen versorgt. Es bestand kein signifikanter Unterschied zwischen den Gruppen (*p*_ISS_ 0,311, *p*_DRG_ 0,456). Es entstanden Kosten in Höhe von 1.363.949 € (5.287 €/PatientIn).

#### Modul „Sonstige Behandlungskosten“

Für radiologische und nuklearmedizinische Untersuchungen entstanden Kosten in Höhe von 331.157 € (1284 €/PatientIn). Für Laboruntersuchungen und Bereitstellung von Transfusionspräparaten 301.832 € (1170 €), für ärztliche Konsile 46.133 € (179 €) und für die Behandlung durch Ergo‑/PhysiotherapeutInnen sowie LogopädInnen entstanden Kosten in Höhe von 76.835 € (298 €) (anteilige Verteilung: Abb. im Zusatzmaterial online: ESM10: Detaillierte Methode, weitere Ergebnisse, weiterführende Literatur).

#### Modul „Overhead“

Die Arbeitsbelastung des Trauma-Leaders für die Annahme von Schwerverletzten und Organisation externer Zuverlegung betrug 54 min/Tag. Daraus resultieren Arbeitskosten von 30.349 € (118 €/PatientIn). Des Weiteren ergeben sich Kosten durch Dokumentation und Zertifizierung von 125.223 € (485 €). Insgesamt entstanden Kosten von 155.572 € (603 €).

#### Gesamtkosten

**Figure 4:**
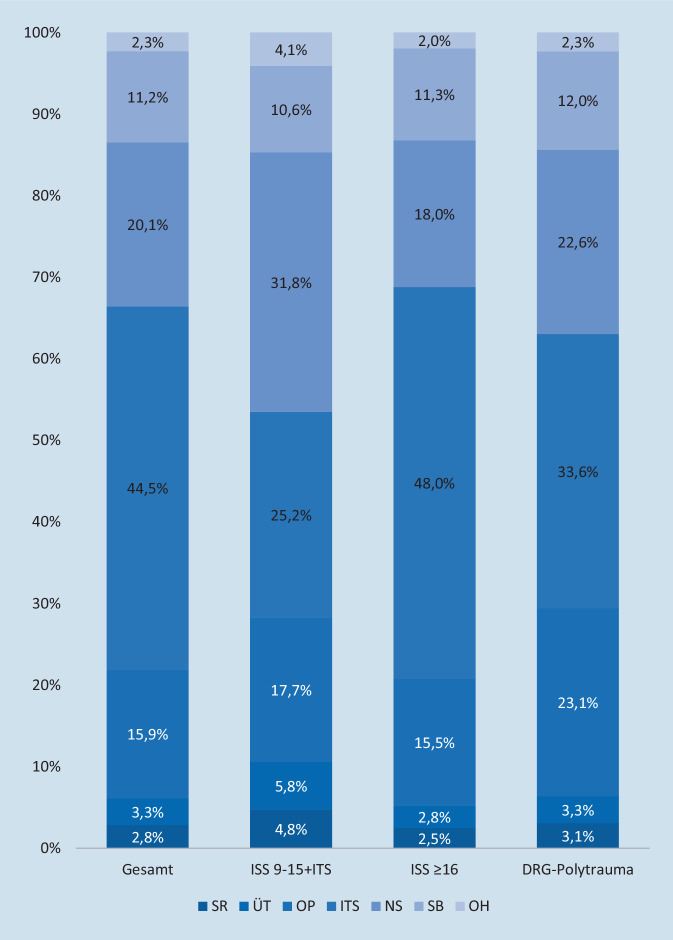

Die Kosten des Moduls „Schockraum“ (1556–1656 €/PatientIn) unterschieden sich in den Subgruppen nicht wesentlich. Die Kosten der Module „OP“ (2609–6018 €), „Intensivstation“ (3705–14.759 €), „Normalstation“ (4681–5903 €) und „Sonstige Behandlungskosten“ (1561–3460 €) unterscheiden sich deutlich. Die Kosten im Modul „Overhead“ lagen bei 603 €/PatientIn. Die Gesamtkosten summierten sich auf 6.773.014 € (26.252 €). Die Kosten der Subgruppen betragen: „ISS 9–15 + ITS“ 14.715 €/PatientIn, „ISS ≥ 16“ 30.718 € und „DRG-Polytrauma“ 26.102 €. Mit 44,5 % macht die intensivmedizinische Behandlung den größten Anteil der Gesamtkosten aus (Abb. 4).

#### Kostenschätzer im TraumaRegister DGU®

Entsprechend der Multiplikatormethode entstanden im Durchschnitt Kosten bei der Versorgung der schwer verletzten PatientInnen in Höhe von 6.811.194 € (26.400 €/PatientIn).

#### Gesamterlöse und Vergleich

**Figure 5:**
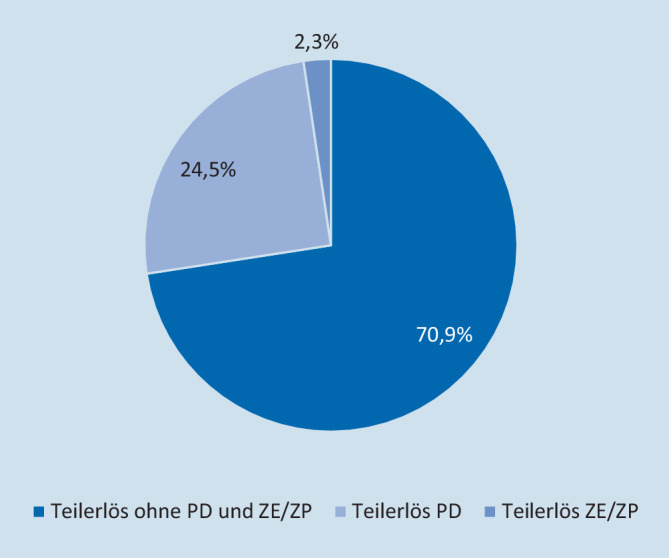

Die simulierten Erlöse des UKL lagen bei 5.261.526 € (20.394 €/PatientIn). In den Subgruppen: „ISS 9–15 + ITS“ 23.902 €/PatientIn, „ISS ≥ 16“ 19.035 € und „DRG-Polytrauma“ 24.120 €. Ein Anteil von 24,5 % der Erlöse entfällt auf die 2020 ausgegliederten Pflegepersonalkosten und 2,3 % auf Zusatzentgelte (Abb. 5).

Im Vergleich besteht eine deutliche Differenz zwischen den Gesamtkosten und den Gesamterlösen. Das Defizit beträgt 1.511.488 € (5858 €).

## Diskussion

Ziel dieser Studie war eine aktuelle Kosten-Erlös-Analyse der Behandlung schwer verletzter PatientInnen, basierend auf einem erweiterten Model nach Pape et al., welches auch Kosten der Organisation von Verlegungen, Zusatzkosten durch „Übertriagierung“ und den Mehraufwand durch Dokumentationspflichten und Zertifizierungskosten berücksichtigt sowie die Erlösstruktur des neu eingeführten aG-DRG-Systems [18].

Am UKL wird für das Jahr 2020 ein Defizit von 1.511.488 € durch die Versorgung von schwer verletzter PatientInnen kalkuliert. Allerdings bestehen deutliche Unterschiede in den Subgruppen; es entstehen Gewinne von 9187 €/PatientIn der Gruppe „ISS 9–15 + ITS“ und Defizite von 11.683 € bzw. 1981 € in den Gruppen „ISS ≥ 16“ bzw. „DRG-Polytrauma“. Eine Subgruppenanalyse der Polytrauma-DRG finden Sie im Zusatzmaterial online: Tab. 9.

Ab dem Jahr 2020 wird der Fallpauschalenkatalog um den Pflegeerlöskatalog erweitert und die Bezeichnung auf „aG-DRG-Katalog“ geändert. Die Höhe des Pflegeentgeltes pro Tag berechnet sich auf Basis eines krankenhausindividuellen Pflegeentgeltwertes. Diese Änderung ist in den Erlösen 2020 bereits berücksichtigt. Ein Erlösanteil von fast 25 % entfällt dabei auf die ausgegliederten Pflegepersonalkosten.

Die Ergebnisse zeigen, dass die Versorgung von Schwerverletzten 17 Jahre nach Einführung des G‑DRG-Systems weiterhin deutlich defizitär erfolgt. Damit bestätigt sich der positive Trend in der Vergütung von Polytraumata der vorhergegangenen Untersuchungen nicht [12–17]. Die jährliche Kostensteigerung 2017–2020 beträgt 4,4 %, hingegen wachsen die Erlöse nur um 1,1 % pro Jahr (vgl. 2,9 % Kostensteigerung in deutschen Krankenhäusern pro Behandlungsfall (Zusatzmaterial online: Tab. 2)) [19–22]. Hierdurch droht sich das Defizit weiter zu vergrößern.

Alleine durch den Personalaufwand bei der Versorgung übertriagierter PatientInnen sind im Modul Schockraum Kosten in Höhe von 220.284 € entstanden, was einem Anteil von 3,3 % der Gesamtbehandlungskosten entspricht. Dies liegt an den weitgefassten Kriterien zur Aktivierung des Schockraumalarms [23]. Hierdurch wird zwar eine große Sensitivität, aber keine gute Spezifität erreicht, sodass 69 % aller über den Schockraum aufgenommenen Patienten kein Polytrauma im definierenden Sinne darstellen. Ausstehend bleibt dabei die Möglichkeit zur Personalkostenreduktion durch eine gestaffelte Schockraumaktivierung, wie in der 3. erweiterten Auflage des Weißbuch der DGU für überregionale Traumazentren gefordert und am UKL zwischenzeitlich umgesetzt („gelber Alarm“ für wache und kreislaufstabile PatientInnen mit einem reduzierten Kernteam und „roter Alarm“ für bewusstlose oder kreislaufinstabile PatientInnen mit allen Teammitgliedern) [24].

Aktuell sind in den Fallpauschalen des DRG-Systems die Versorgung der übertriagierten PatientInnen und die Kosten für Dokumentation, Zertifizierung und Organisation nicht hinreichend abgebildet. Auch wenn man den Versorgungsaufwand ohne diese Kosten betrachtet, ergibt sich ein Defizit von 1.135.632 € (4402 €/PatientIn).

Die Möglichkeiten zur Reduktion laufender Krankenhauskosten in der Versorgung von schwer verletzten PatientInnen sind begrenzt. Taheri et al. zeigten, dass Einsparungen im Bereich der intensivmedizinischen Versorgung von schwer verletzten PatientInnen im Bereich der variablen und damit direkt beeinflussbaren Kosten die geringste Summe der Ausgaben darstellen [25, 26]. Jedoch konnten im Rahmen dieser Studien Einsparpotenziale von ca. 25–35 % im Rahmen der ärztlichen Behandlung erzielt werden. Der größte Einflussfaktor sind die intensivmedizinischen Kosten (3.011.891 € bzw. 44,5 % der Gesamtkosten). Hier sollten medizinisch sinnvolle Möglichkeiten zur Effizienzsteigerung konsequent umgesetzt werden. Die aktuelle Entwicklung der intensivmedizinischen Versorgung schwer verletzter PatientInnen zeigt beispielsweise einen, medizinisch und wirtschaftlich begrüßenswerten, Rückgang der Beatmungsdauer auf Intensivstationen [27]. Zusätzlich kommt es in den letzten Jahren zunehmend zu einer Reduktion der Gesamtverweildauer im Krankenhaus, was die Kosten für den Verlauf auf der Normalstation ebenfalls reduziert [27].

Sollte sich keine Verbesserung der Kosten‑/Erlösstruktur bei der Polytraumaversorgung ergeben, bleibt zu befürchten, dass durch Selektionieren der angenommenen PatientInnen oder sogar durch Schließungen von Traumazentren versucht werden könnte, erlösoptimiert zu behandeln.

Die Entscheidung zur Versorgung schwer verletzter PatientInnen darf keine finanziell beeinflusste Überlegung sein. Sie ist Teil des Versorgungsauftrags aller lokalen, regionalen und überregionalen Traumazentren. Folglich bleibt es der Politik überlassen, durch eine adäquate Finanzierung von Traumazentren die Überlebensraten und das funktionelle Outcome der Patienten auf dem hohen Niveau zu halten, das wir in Deutschland erreicht haben [28, 29] – nicht nur aufgrund ethischer Bedenken, sondern auch weil der volkswirtschaftliche Schaden durch Verlust von Arbeitskraftpotenzial und durch Aufwendungen der gesetzlichen Unfallversicherung wesentlich höher ist.

Die Errechnung der in dieser Studie genannten Kosten basiert auf Schätzungen anhand der den AutorInnen zur Verfügung stehenden Daten (ausführlicher Methodenteil im Zusatzmaterial online: ESM10: Detaillierte Methode, weitere Ergebnisse, weiterführende Literatur). Für einige Positionen mussten hier Annäherungen getroffen werden, die zudem spezifisch für das Universitätsklinikum Leipzig sind und regional unterschiedlich sein können (z. B. Miete). In der Gesamtheit ist der Ansatz zur Kostenschätzung jedoch detaillierter als die der vorangegangenen Studien [12–17].

Für die betriebswirtschaftliche Betrachtung muss zwischen patientenunabhängigen Kosten (Fixkosten, z. B. Infrastrukturkosten des Schockraums) und patientenabhängigen Kosten (variablen Kosten, z. B. Implantatkosten bei Operationen) unterschieden werden. Um für erbrachte medizinische Behandlungen leistungsgerechte Erlöse mit Fallpauschalen zu vergüten, müsste man die Grenzkosten der Behandlung von schwer verletzten PatientInnen betrachten (Kosten, die durch die Versorgung zusätzlicher Fälle entstehen). Limitierend werden in dieser Studie aber kalkulierte Durchschnittskosten der behandelten PatientInnen untersucht. Diese Limitation relativiert sich, wenn man die ebenfalls retrospektive Datenerhebung des InEK zur Erhebung des Relativgewichts der einzelnen DRG aus den jährlichen Kostendaten der Kalkulationskrankenhäuser in die Überlegung miteinbezieht.

## Fazit für die Praxis

Im angestellten Vergleich von Behandlungskosten für schwer verletzte PatientInnen und der Vergütung nach aG-DRG an einem universitären Maximalversorger und überregionalen Traumazentrum zeigt sich ein erhebliches betriebswirtschaftliches Defizit auf. Die Ergebnisse dieser Studie sollen als Diskussionsgrundlage für weitere und unbedingt notwendige Verhandlungen bei der Kalkulation der Fallpauschalen im deutschen DRG-System hin zu einer gerechteren Entlohnung dieser komplexen Behandlungsfälle und Abdeckung der Vorhaltekosten dienen, sollten aber auch zu Überlegungen bezüglich einer kosteneffizienteren Versorgung innerhalb der Traumazentren motivieren.

## Supplementary Information













