# Supplementary material for: Is polytrauma treatment in deficit in the aG-DRG system?
Source: Unfallchirurg. 2021 Jun 8;125(4):305–12. [Article in German] doi: 10.1007/s00113-021-01015-5 (PMC8940839; doi:10.1007/s00113-021-01015-5)
Supplement: Supplementary file 1 [file 113_2021_1015_MOESM1_ESM.pdf]

DRG	Jahr	Bewertungsrelation bei Hauptabteilung	Mittlere Verweildauer	Untere Grenzverweildauer		Obere Grenzverweildauer		Pflegeerlös Bewertungs-relation/Tag
				Erster Tag mit Abschlag	Bewertungs-relation/Tag	Erster Tag zus. Entgelt	Bewertungs-relation/Tag	
**W01B**	Polytrauma mit Beatmung > 72 Stunden oder komplexen Eingriffen oder IntK > 392 / 368 / 552 Aufwandspunkte, ohne Frührehabilitation, mit Beatmung > 263 Stunden oder mit komplexer Vakuumbehandlung oder mit IntK > 588 / 552 / -							
	2020	10,454	25,9	8	0,773	44	0,268	2,7119
	2019	14,288	31,6	10	0,941	50	0,331	Seit aG-DRG 2020
	2018	14,563	31,7	10	0,973	50	0,338	
	2017	14,648	31,7	10	0,996	50	0,346	
**W01C**	Polytrauma mit Beatmung > 72 Stunden oder komplexen Eingriffen oder IntK > 392 / 368 / 552 Aufwandspunkte, ohne Frührehabilitat							
	2020	3,968	13,8	4	0,609	28	0,222	2,2879
	2019	7,107	18,4	5	0,934	34	0,305	Seit aG-DRG 2020
	2018	7,852	18,5	5	0,99	35	0,32	
	2017	8,051	21	6	0,87	38	0,298	
**W02A**	Polytrauma mit anderen komplexen Eingriffen mit komplizierender Konstellation oder Eingriffen an mehreren Lokalisationen oder mi							
	2020	7,974	25,3	7	0,498	43	0,11	1,6205
	2019	8,989	23,7	7	0,644	42	0,152	Seit aG-DRG 2020
	2018	8,142	22,9	7	0,556	40	0,136	
	2017	7,782	22	6	0,613	39	0,136	
**W02B**	Polytrauma mit anderen komplexen Eingriffen ohne komplizierende Konstellation, ohne Eingriffe an mehreren Lokalisationen, ohne i							
	2020	4,636	17,3	5	0,409	32	0,099	1,3987
	2019	5,615	18,1	5	0,572	33	0,133	Seit aG-DRG 2020
	2018	4,671	16,8	5	0,489	31	0,123	
	2017	4,853	17,3	5	0,509	33	0,123	
**W04A**	Polytrauma mit anderen Eingriffen oder Beatmung > 24 Stunden, mit komplizierender Konstellation oder Eingriffen an mehreren Loka							
	2020	5,22	18,2	5	0,464	33	0,107	1,5646
	2019	6,641	19,9	6	0,573	35	0,141	Seit aG-DRG 2020
	2018	6,846	21,1	6	0,583	37	0,136	
	2017	6,093	19,9	6	0,52	36	0,128	
**W04B**	Polytrauma mit anderen Eingriffen oder Beatmung > 24 Stunden, ohne komplizierende Konstellation, ohne Eingriffe an mehreren Lok							
	2020	3,087	14,5	4	0,375	28	0,091	1,213
	2019	3,729	14,5	4	0,523	28	0,126	Seit aG-DRG 2020
	2018	3,688	14,7	4	0,522	29	0,124	
	2017	3,769	14,8	4	0,537	28	0,127	
**W04C**	Polytrauma mit anderen Eingriffen oder Beatmung > 24 Stunden, ohne komplizierende Konstellation, ohne Eingriffe an mehreren Lok							
	2020	2,02	11,5	3	0,327	23	0,079	1,1964
	2019	2,23	8,9	2	0,519	16	0,123	Seit aG-DRG 2020
	2018	Seit G-DRG 2019						
	2017							
**W36Z**	Intensivmedizinische Komplexbehandlung > 784 / 828 / 828 Aufwandspunkte bei Polytrauma oder Polytrauma mit Beatmung oder Kra							
	2020	11,477	29,7	9	0,841	48	0,283	3,174
	2019	17,028	30,8	9	1,35	49	0,438	Seit aG-DRG 2020
	2018	16,423	32	10	1,204	50	0,415	
	2017	16,423	32	10	1,204	50	0,415	
**W60Z**	Polytrauma, verstorben < 5 Tage nach Aufnahme, ohne komplizierende Konstellationen, ohne Beatmung > 24 Stunden, ohne komplex							
	2020	0,624	1,2	Nicht vorgesehen				1,5648
	2019	1,804	1,6					Seit aG-DRG 2020
	2018	2,059	1,6					
	2017	2,058	1,5					
**W61A**	Polytrauma ohne signifikante Eingriffe mit komplizierender Diagnose oder mit intensivmedizinischer Komplexbehandlung > 196 / 184							
	2020	1,725	11,2	3	0,418	24	0,105	1,4328
	2019	2,072	10	2	0,666	21	0,14	Seit aG-DRG 2020
	2018	2,127	10,2	2	0,689	22	0,142	
	2017	2,114	10,6	3	0,516	22	0,136	
**W61B**	Polytrauma ohne signifikante Eingriffe, ohne komplizierende Diagnose, ohne intensivmedizinische Komplexbehandlung > 196 / 184 / -							
	2020	1,161	8,7	2	0,374	18	0,09	1,0873
	2019	1,656	9,5	2	0,537	21	0,118	Seit aG-DRG 2020
	2018	1,737	9,9	2	0,565	21	0,12	
	2017	1,631	9,2	2	0,53	20	0,121	

MDC 21A Polytrauma.
Auszüge der Fallpauschalenkataloge 2017-2020 des InEKs (4–7). Bezeichnungen entsprechend des Fallpauschalenkatalogs 2020.
