# Supplementary material for: Is polytrauma treatment in deficit in the aG-DRG system?
Source: Unfallchirurg. 2021 Jun 8;125(4):305–12. [Article in German] doi: 10.1007/s00113-021-01015-5 (PMC8940839; doi:10.1007/s00113-021-01015-5)
Supplement: Supplementary file 2 [file 113_2021_1015_MOESM2_ESM.pdf]

Jahr	Kosten je Behandlungsfall	Personalkosten im Pflegedienst	Personalkosten im ärztlichen	Sachkosten
**2020***	5.120 €	21.088 Mio. €	24.363 Mio. €	44,4 Mrd. €
**2019***	4.974 €	20.424 Mio. €	22.980 Mio. €	42,5 Mrd. €
**2018***	4.833 €	19.781 Mio. €	21.675 Mio. €	40,8 Mrd. €
**Gemittelte Steigerung pro Jahr**	**2,93%**	**3,25%**	**6,02%**	**4,29%**
**2017**	4.695 €	19.158 Mio. €	20.445 Mio. €	39,1 Mrd. €
**2016**	4.497 €	18.359 Mio. €	19.512 Mio. €	37,9 Mrd. €
**2015**	4.378 €	17.751 Mio. €	18.537 Mio. €	36,2 Mrd. €
**2014**	4.239 €	17.098 Mio. €	17.578 Mio. €	35,0 Mrd. €
**2013**	4.152 €	16.510 Mio. €	16.671 Mio. €	33,8 Mrd. €
**2012**	4.060 €	16.181 Mio. €	15.768 Mio. €	32,6 Mrd. €
**2011**	3.960 €	15.707 Mio. €	14.729 Mio. €	31,7 Mrd. €
**2010**	3.862 €	15.150 Mio. €	13.901 Mio. €	30,3 Mrd. €
**2009**	3.771 €	14.805 Mio. €	13.048 Mio. €	29,2 Mrd. €
**2008**	3.609 €	14.232 Mio. €	12.117 Mio. €	27,3 Mrd. €
**2007**	3.518 €	13.916 Mio. €	11.400 Mio. €	25,7 Mrd. €

Kostenentwicklung in deutschen Krankenhäusern.
Gemittelte prozentuale Steigerung anhand der Daten von 2007-2017.
* Prognostizierte Kosten
