# Supplementary material for: Is polytrauma treatment in deficit in the aG-DRG system?
Source: Unfallchirurg. 2021 Jun 8;125(4):305–12. [Article in German] doi: 10.1007/s00113-021-01015-5 (PMC8940839; doi:10.1007/s00113-021-01015-5)
Supplement: Supplementary file 3 [file 113_2021_1015_MOESM3_ESM.pdf]

Dienstgruppe	SL 2017	SL 2018*	SL 2019*	SL 2020*
Ärztlicher Dienst	63,26 €	67,07 €	71,11 €	75,39 €
Pflegedienst	32,24 €	33,29 €	34,38 €	35,49 €

Stundenlohn [SL] pro Mitarbeiter des ärztlichen Dienstes und Pflegedienstes.
* Prognostizierte Kosten
