# Supplementary material for: Is polytrauma treatment in deficit in the aG-DRG system?
Source: Unfallchirurg. 2021 Jun 8;125(4):305–12. [Article in German] doi: 10.1007/s00113-021-01015-5 (PMC8940839; doi:10.1007/s00113-021-01015-5)
Supplement: Supplementary file 4 [file 113_2021_1015_MOESM4_ESM.pdf]

	2017*	2018*	2019	2020*
**Betriebskosten**	1.509,01 €	1.573,74 €	1.641,26 €	1.711,67 €
**Reinigung**	7.412,67 €	7.730,67 €	8.062,32 €	8.408,19 €
**Energie**	2.012,85 €	2.099,20 €	2.189,26 €	2.283,17 €
**Medizintechnik**	12.675,73 €	13.219,52 €	13.786,64 €	14.378,08 €
**Kaltmiete**	6.088,98 €	6.350,20 €	6.622,62 €	6.906,73 €
**Gesamtkosten**	30.524,32 €	31.833,81 €	33.199,48 €	34.623,74 €
**pro SR-Patient**	**36,60 €**	**38,17 €**	**39,81 €**	**41,52 €**

Jährliche Infrastrukturkosten des chirurgischen Schockraums am UKL. Betriebskosten inklusive Gebäudetechnik und -wartung. Reinigung laut Subunternehmen 14 €/m2 Monat. Energie (Strom, Wasser, Wärme). Medizintechnik beinhaltet Reparatur, Material, Wartung und Prüfung von 1,0 Patientenmonitor, 1,0 Narkose-Beatmungsgerät, 2,0 Infusionsspritzenpumpen, 0,5 Ultraschallgerät, 1,0 Defibrillator, 1,0 OP-Leuchte, 1,0 Patientenstretcher, 1,0 Endoskop, 0,5 BGA-Gerät, 1,0 EKG-Gerät. Kaltmiete entspricht der Angesetzten Miete des Medizinischen Versorgungszentrums am UKL. * Prognostizierte Kosten.
