# Supplementary material for: Is polytrauma treatment in deficit in the aG-DRG system?
Source: Unfallchirurg. 2021 Jun 8;125(4):305–12. [Article in German] doi: 10.1007/s00113-021-01015-5 (PMC8940839; doi:10.1007/s00113-021-01015-5)
Supplement: Supplementary file 5 [file 113_2021_1015_MOESM5_ESM.pdf]

Kostenposition	anteilige Wochenstunden [1]	Jahresbetrag Wert in €	Anteiliger Jahreswert in €[2]
**Personalkosten**			
- Dokumentationskraft	40 / 40 h	42.565,30 €	42.565,30 €
- Assistenzarzt	10 / 42 h	93.305,88 €	22.215,69 €
- Oberarzt	4 / 42 h	158.500,73 €	15.095,31 €
**Investitions- und Sachaufwand**			
- Jahresbeitrag TraumaRegister DGU®	jährlich	595,00 €	595,00 €
- Zertifizierungskosten	3-jährlich	5.950,00 €	1.983,33 €
- begleitende Reisekosten	3-jährlich	260,00 €	86,67 €
**Sonstige Kosten[3]**	jährlich		25.971,63 €
**Gesamtaufwand im Jahr 2017**			**108.512,92 €**

Kalkulation des Mehraufwands des Traumazentrum am Beispiel 2017.

[1]Anteilige Wochenstundenzahl bei Personal. [2]Inkl. Tariferhöhung 2017 bei Personalkosten.

[3]Sonstige Kosten als Oberbegriff für beispielsweise Raum-, Unterhaltungskosten, Organisationskosten, Inanspruchnahme Klinikdirektor und Sekretariat.
