# Supplementary material for: Is polytrauma treatment in deficit in the aG-DRG system?
Source: Unfallchirurg. 2021 Jun 8;125(4):305–12. [Article in German] doi: 10.1007/s00113-021-01015-5 (PMC8940839; doi:10.1007/s00113-021-01015-5)
Supplement: Supplementary file 6 [file 113_2021_1015_MOESM6_ESM.pdf]

	2010	2011	2012	2013	2014	2015	2016	2017	2018	2019	2020*
**LBFW Sachsen**	2864	2884	2958	3013	3117	3191	3278	3342	3439	3529	3612
**BBFW**	2936	2964	2992	3068	3157	3231	3312	3376	3467	3545	3625

Basisfallwerte 2010-2020.
LBFW - Landesbasisfallwert, BBFW - Bundesbasisfallwert. Mittlere Steigerungsrate 2010-2019: LBFW Sachsen 2,35 %, BBFW 2,27 %.
