# Supplementary material for: Is polytrauma treatment in deficit in the aG-DRG system?
Source: Unfallchirurg. 2021 Jun 8;125(4):305–12. [Article in German] doi: 10.1007/s00113-021-01015-5 (PMC8940839; doi:10.1007/s00113-021-01015-5)

	Kostenpunkte		Gruppe "Gesamt"	pro Patient "Gesamt"	Gruppe "ISS 9-15 + ITS"	pro Patient "ISS 9-15 + ITS"	Gruppe "ISS ≥ 16"	pro Patient "ISS ≥ 16"	Gruppe "DRG-Polytrauma"	pro Patienten "DRG-Polytrauma"
Anzahl der Patienten		n	258		72		186		59	
Relativgewicht 2017*			1447,4	5,8	485,9	6,7	961,4	5,5	398,0	6,7
Relativgewicht 2020*			1116,1	4,5	367,3	5,1	748,8	4,3	303,9	5,2
Schockraum	Dauer	min	9631	37,3	2544	35,3	7087	38,1	2368	40,1
	Personal ÄD 2017	€	93.355 €	362 €	23.750 €	330 €	69.605 €	374 €	24.055 €	408 €
	Personal ÄD 2020 (1)	€	111.818 €	433 €	28.448 €	395 €	83.370 €	448 €	28.813 €	488 €
	Personal PD 2017	€	14.637 €	57 €	3.895 €	54 €	10.742 €	58 €	3.551 €	60 €
	Personal PD 2020 (2)	€	16.111 €	62 €	4.287 €	60 €	11.824 €	64 €	3.909 €	66 €
	Umverteilung Personalkosten 2017	€	186.977 €	725 €	52.180 €	725 €	134.798 €	725 €	42.758 €	725 €
	Umverteilung Personalkosten 2020 (1 u. 2)	€	220.284 €	854 €	61.475 €	854 €	158.809 €	854 €	50.375 €	854 €
	Infrastruktur 2017	€	30.761 €	119 €	8.518 €	118 €	22.243 €	120 €	6.980 €	118 €
	Infrastruktur 2020 (3)	€	34.623 €	134 €	9.662 €	134 €	24.961 €	134 €	7.918 €	134 €
	Verbrauchsgüter 2017	€	25.800 €	100 €	7.200 €	100 €	18.600 €	100 €	5.900 €	100 €
	Verbrauchsgüter 2020 (3)	€	29.265 €	113 €	8.167 €	113 €	21.098 €	113 €	6.692 €	113 €
	**Modulkosten 2017**	**€**	**351.530 €**	**1.363 €**	**95.544 €**	**1.327 €**	**255.987 €**	**1.376 €**	**83.245 €**	**1.411 €**
	**Modulkosten 2020**	**€**	**412.101 €**	**1.597 €**	**112.039 €**	**1.556 €**	**300.062 €**	**1.613 €**	**97.707 €**	**1.656 €**
OP	Anzahl der OPs	n	457	1,8	89	1,2	368	2,0	156	2,6
	SN-Zeit	min	37531	145,5	7184	99,8	30347	163,2	13491	228,7
	Anästhesie 2017	€	313.173 €	1.214 €	56.310 €	782 €	256.863 €	1.381 €	112.349 €	1.904 €
	Anästhesie 2020 (3)	€	355.232 €	1.377 €	63.873 €	887 €	291.359 €	1.566 €	127.438 €	2.160 €
	Peronsal ÄD Chirurgie 2017	€	122.994 €	477 €	22.426 €	311 €	100.568 €	541 €	46.457 €	787 €
	Peronsal ÄD Chirurgie 2020 (1)	€	146.570 €	568 €	26.725 €	371 €	119.846 €	644 €	55.363 €	938 €
	Peronsal PD Chirurgie 2017	€	61.318 €	238 €	11.276 €	157 €	50.043 €	269 €	22.839 €	387 €
	Peronsal PD Chirurgie 2020 (2)	€	67.493 €	262 €	12.411 €	172 €	55.082 €	296 €	25.139 €	426 €
	Material und Implantate 2017	€	437.588 €	1.696 €	73.439 €	1.020 €	364.149 €	1.958 €	127.209 €	2.156 €
	Material und Implantate 2020 (3)	€	496.356 €	1.924 €	83.302 €	1.157 €	413.054 €	2.221 €	144.293 €	2.446 €
	Infrastruktur 2017	€	6.958 €	27 €	1.332 €	18 €	5.626 €	30 €	2.501 €	42 €
	Infrastruktur 2020 (3)	€	7.893 €	31 €	1.511 €	21 €	6.382 €	34 €	2.837 €	48 €
	**Modulkosten 2017**	**€**	**942.031 €**	**3.651 €**	**164.783 €**	**2.289 €**	**777.248 €**	**4.179 €**	**311.355 €**	**5.277 €**
	**Modulkosten 2020**	**€**	**1.073.544 €**	**4.161 €**	**187.821 €**	**2.609 €**	**885.723 €**	**4.762 €**	**355.069 €**	**6.018 €**
Intensiv-station	Behandlungstage Intensivstation	d	2032	7,9	196	2,7	1837	9,9	419	7,1
	Beatmungstage	d	770	3,0	51	0,7	719	3,9	127	2,1
	**Modulkosten 2017**	**€**	**2.655.286 €**	**10.292 €**	**235.205 €**	**3.267 €**	**2.420.080 €**	**13.011 €**	**456.526 €**	**7.738 €**
	**Modulkosten 2020 (3)**	**€**	**3.011.891 €**	**11.674 €**	**266.793 €**	**3.705 €**	**2.745.098 €**	**14.759 €**	**517.838 €**	**8.777 €**
Normal-station	Behandlungstage	d	2654	10,3	662	9,2	1992	10,7	673	11,4
	**Modulkosten 2017**	**€**	**1.202.458 €**	**4.661 €**	**297.126 €**	**4.127 €**	**905.332 €**	**4.867 €**	**307.017 €**	**5.204 €**
	**Modulkosten 2020 (3)**	**€**	**1.363.949 €**	**5.287 €**	**337.030 €**	**4.681 €**	**1.026.919 €**	**5.521 €**	**348.249 €**	**5.903 €**

Sonstige Behandlungskosten	Radiologie und Nuklearmedizin 2017	€	291.948 €	1.132 €	51.652 €	717 €	240.296 €	1.292 €	65.895 €	1.117 €
	Radiologie und Nuklearmedizin 2020 (3)	€	331.156 €	1.284 €	58.589 €	814 €	272.567 €	1.465 €	74.744 €	1.267 €
	C/M/V/P/I 2017	€	120.066 €	465 €	17.895 €	249 €	102.171 €	549 €	24.608 €	417 €
	C/M/V/P/I 2020 (3)	€	136.190 €	528 €	20.298 €	282 €	115.892 €	623 €	27.913 €	473 €
	Transfusionspräparate	n	1034	4,0	18	0,3	1016	5,5	473	8,0
	Transfusionsmedizin 2017	€	146.029 €	566 €	7.682 €	107 €	138.348 €	744 €	44.769 €	759 €
	Transfusionsmedizin 2020 (3)	€	165.641 €	642 €	8.713 €	121 €	156.928 €	844 €	50.782 €	861 €
	Ärztliche Konsile 2017	€	38.712 €	150 €	10.628 €	148 €	28.084 €	151 €	10.404 €	176 €
	Ärztliche Konsile 2020 (1)	€	46.133 €	179 €	12.666 €	176 €	33.467 €	180 €	12.398 €	210 €
	P/E/L 2017	€	69.806 €	271 €	10.994 €	153 €	58.812 €	316 €	17.912 €	304 €
	P/E/L 2020 (2)	€	76.835 €	298 €	12.101 €	168 €	64.735 €	348 €	19.715 €	334 €
	**Modulkosten 2017**	**€**	**666.561 €**	**2.584 €**	**98.851 €**	**1.373 €**	**567.710 €**	**3.052 €**	**163.587 €**	**2.773 €**
	**Modulkosten 2020**	**€**	**755.956 €**	**2.930 €**	**112.367 €**	**1.561 €**	**643.589 €**	**3.460 €**	**185.553 €**	**3.145 €**
Overhead	Z/D/O 2017	€	108.513 €	421 €	30.283 €	421 €	78.230 €	421 €	24.815 €	421 €
	Z/D/O 2020 (4)	€	125.223 €	485 €	34.946 €	485 €	90.277 €	485 €	28.636 €	485 €
	Kosten Traumahandy 2017	€	25.467 €	99 €	7.107 €	99 €	18.360 €	99 €	5.824 €	99 €
	Kosten Traumahandy 2020 (1)	€	30.349 €	118 €	8.469 €	118 €	21.880 €	118 €	6.940 €	118 €
	**Modulkosten 2017**	**€**	**133.980 €**	**519 €**	**37.390 €**	**519 €**	**96.590 €**	**519 €**	**30.639 €**	**519 €**
	**Modulkosten 2020**	**€**	**155.572 €**	**603 €**	**43.415 €**	**603 €**	**112.156 €**	**603 €**	**35.577 €**	**603 €**
Gesamt	**Gesamtkosten 2017**	**€**	**5.951.846 €**	**23.069 €**	**928.899 €**	**12.901 €**	**5.022.947 €**	**27.005 €**	**1.352.370 €**	**22.922 €**
	**Gesamtkosten 2020**	**€**	**6.773.014 €**	**26.252 €**	**1.059.467 €**	**14.715 €**	**5.713.547 €**	**30.718 €**	**1.539.992 €**	**26.102 €**
	Kostenschätzer 2017	€	6.245.698 €	24.208 €	1.886.547 €	26.202 €	4.359.151 €	23.436 €	1.500.816 €	25.438 €
	Kostenschätzer 2020	€	6.811.194 €	26.400 €	2.057.358 €	28.574 €	4.753.836 €	25.558 €	1.636.703 €	27.741 €
	**Gesamterlös 2017**	**€**	**5.094.492 €**	**19.746 €**	**1.675.621 €**	**23.273 €**	**3.418.871 €**	**18.381 €**	**1.377.611 €**	**23.349 €**
	**Gesamterlös 2020**	**€**	**5.261.526 €**	**20.394 €**	**1.720.960 €**	**23.902 €**	**3.540.567 €**	**19.035 €**	**1.423.096 €**	**24.120 €**
	Teilerlös 2020 ohne Pflege und ZE/ZP	€	3.729.592 €	14.456 €	1.227.296 €	17.046 €	2.502.296 €	13.453 €	1.015.510 €	17.212 €
	Teilerlös 2020 Pflege	€	1.289.213 €	4.997 €	446.108 €	6.196 €	843.105 €	4.533 €	363.588 €	6.163 €
	Teilerlös 2020 Zusatzentgelte	€	121.143 €	470 €	35.398 €	492 €	85.744 €	461 €	31.840 €	540 €
	**2017**	€	**-857.355 €**	**-3.323 €**	**746.722 €**	**10.371 €**	**-1.604.077 €**	**-8.624 €**	**25.241 €**	**428 €**
	**2020**	€	**-1.511.488 €**	**-5.858 €**	**661.493 €**	**9.187 €**	**-2.172.981 €**	**-11.683 €**	**-116.895 €**	**-1.981 €**

Ausführliche Darstellung der Tabelle 2. Kosten und Erlöse im Jahr 2017 und 2020 sowie in den einzelnen Subgruppen. *Die zehn Patienten mit der Diagnose B61B sind nicht berücksichtigt. Jährliche Steigerungsraten: (1) Personalkosten ÄD: 6,20 %, (2) Personalkosten PD: 3,25 %, (3) Sachkosten: 4,29 %, (4) Z/D/O-Kosten 4,89 %. C/M/V/P/I - Klinische Chemie, Mikrobiologie, Virologie, Pathologie und Immunologie, P/E/L - Physio- / Ergotherapie und Logopädie, Z/D/O - Zertifizierung-, Dokumentations- und Organisationskosten.

	Gesamt	ISS 9-15+ITS	ISS ≥16	-Polytrauma
SR	2,8%	4,8%	2,5%	3,1%
ÜT	3,3%	5,8%	2,8%	3,3%
OP	15,9%	17,7%	15,5%	23,1%
ITS	44,5%	25,2%	48,0%	33,6%
NS	20,1%	31,8%	18,0%	22,6%
SB	11,2%	10,6%	11,3%	12,0%
OH	2,3%	4,1%	2,0%	2,3%

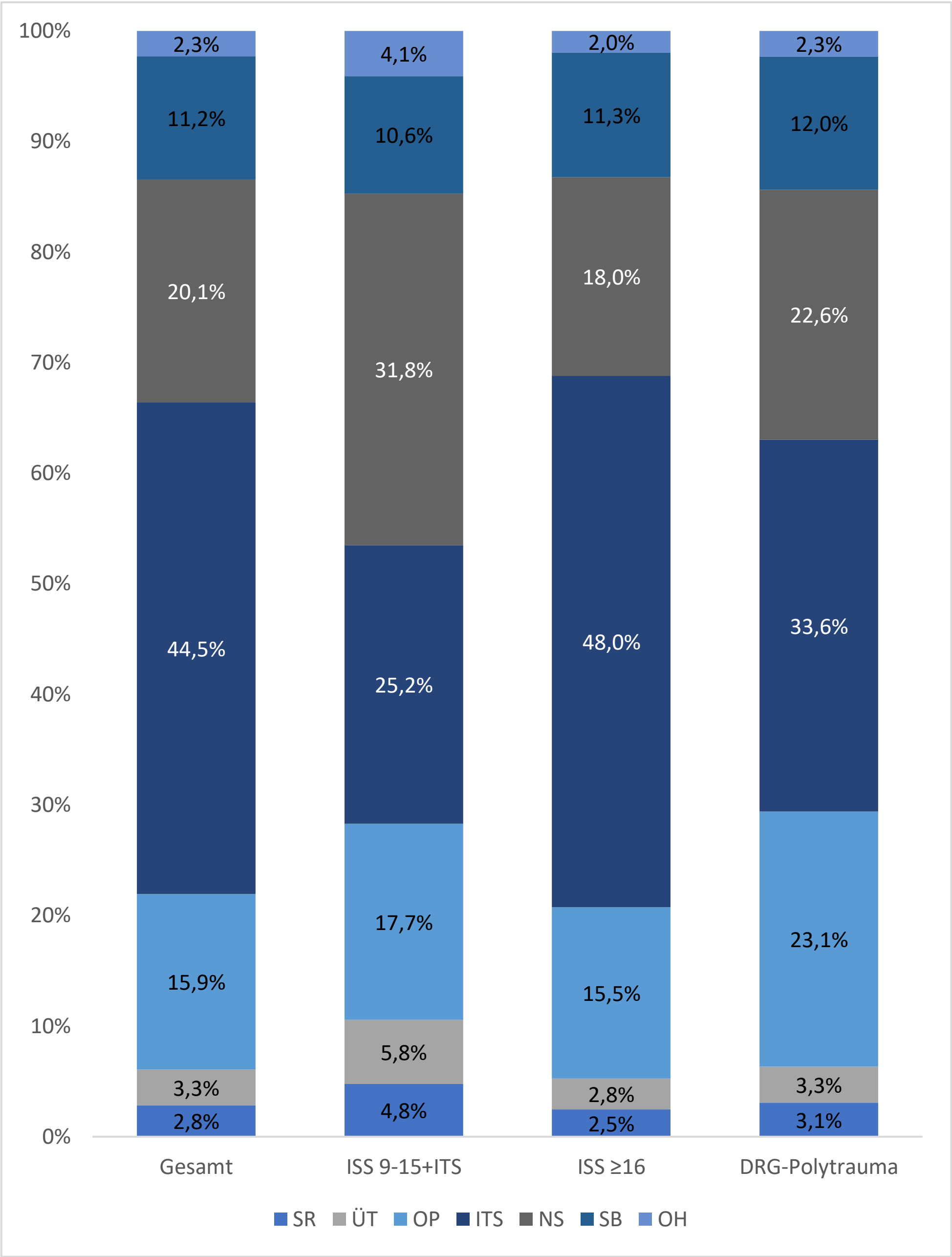

Supplement: Supplementary file 7 [file 113_2021_1015_MOESM7_ESM.pdf]
