# Supplementary material for: Is polytrauma treatment in deficit in the aG-DRG system?
Source: Unfallchirurg. 2021 Jun 8;125(4):305–12. [Article in German] doi: 10.1007/s00113-021-01015-5 (PMC8940839; doi:10.1007/s00113-021-01015-5)
Supplement: Supplementary file 8 [file 113_2021_1015_MOESM8_ESM.pdf]

	**Prädikatorvariable**	**KW 2008**	**KW 2017***	**KW 2018***	**KW 2019***	**KW 2020***
	Anzahl der Tage auf Intensivstation x	1.152 €	1.537 €	1.582 €	1.629 €	1.677 €
+	Anzahl der Tage mit Intubation x	568 €	758 €	780 €	803 €	827 €
+	Anzahl der Tage auf Normalstation x	531 €	709 €	729 €	751 €	773 €
+	Anzahl der gegebenen EKs und FFPs bis zur Aufnahme auf Intensivstation	258 €	344 €	354 €	365 €	375 €
+	Falls schwere Abdominalverletztung (AIS ≥ 3)	2.849 €	3.802 €	3.914 €	4.028 €	4.146 €
+	Falls instabile Beckenverletzung (AIS = 5)	7.505 €	10.016 €	10.310 €	10.612 €	10.923 €
+	Falls schwere Extremitäten oder Beckenverletzung (AIS = 3-4)	2.418 €	3.227 €	3.322 €	3.419 €	3.519 €

Kalkulations der Multiplikatoren des Kostenschätzers im TraumaRegister DGU®. * Prognostizierte Kosten.
