# Supplementary material for: Is polytrauma treatment in deficit in the aG-DRG system?
Source: Unfallchirurg. 2021 Jun 8;125(4):305–12. [Article in German] doi: 10.1007/s00113-021-01015-5 (PMC8940839; doi:10.1007/s00113-021-01015-5)
Supplement: Supplementary file 9 [file 113_2021_1015_MOESM9_ESM.pdf]

DRG	Fälle (n)	Behandlungs-tage pro Fall	Kosten 2017	Gesamterlös 2017	Differenz K/GE 2017	Differenz K/GE 2018	Differenz K/GE 2019	Differenz K/GE 2020
**W01B**	6	49,33	49.214 €	50.298 €	-1.083 €	-1.321 €	-1.778 €	-1.892 €
**W01C**	6	24,33	26.286 €	28.474 €	2.189 €	1.077 €	1.440 €	882 €
**W02A**	4	64,31	75.029 €	89.603 €	14.574 €	13.586 €	15.194 €	13.863 €
**W02B**	8	16,63	19.337 €	15.501 €	-3.836 €	-5.200 €	-5.258 €	-5.817 €
**W04A**	4	8,63	9.373 €	10.180 €	807 €	1.685 €	1.888 €	1.714 €
**W04B**	7	11,71	15.787 €	12.338 €	-3.449 €	-4.336 €	-4.393 €	-4.860 €
**W36Z**	5	35,4	53.722 €	57.551 €	3.829 €	1.484 €	2.240 €	1.122 €
**W60Z**	14	2,07	9.611 €	8.475 €	-1.136 €	-1.441 €	-1.464 €	-1.721 €
**W61A**	2	19,5	13.129 €	7.064 €	-6.065 €	-1.527 €	-6.735 €	-7.191 €
**W61B**	3	10,67	9.928 €	5.450 €	-4.478 €	-4.495 €	-4.633 €	-4.974 €

Subgruppen Analyse der Patienten in der Polytrauma-DRG MCD 21A. K – Kosten; GE – DRG-Gesamterlös.
* Prognostizierte Kosten.
