# Supplementary material for: Is polytrauma treatment in deficit in the aG-DRG system?
Source: Unfallchirurg. 2021 Jun 8;125(4):305–12. [Article in German] doi: 10.1007/s00113-021-01015-5 (PMC8940839; doi:10.1007/s00113-021-01015-5)
Supplement: Supplementary file 10 [file 113_2021_1015_MOESM10_ESM.pdf]

I. Supplement Methode: Kostenberechnung

Kostenentwicklung in deutschen Krankenhäusern

Die Kostenentwicklung, wenn nicht anders angegeben, für die Jahre 2018 bis 2020 bezieht sich auf die Daten des statistischen Bundesamts aus den Jahren 2007 bis 2017. Es wurde ein gleichbleibender Kostenanstieg angenommen (Supplement Tabelle 2) [1–3].

Personalkosten

Die angegebenen Personalkosten basieren auf den realen Personalkosten des UKL im Jahr 2017. In der Arbeitszeitberechnung wird von einem 365-Tage-Jahr mit 30 Urlaubstagen ausgegangen und mit Wochenstunden im Bereich des ärztlichen Dienstes [ÄD] von 42 Stunden und des Pflegedienstes [PD], inklusive der Funktionsdienste und des physiotherapeutischen Dienstes, von 40 Stunden gerechnet. Aufgrund der hohen Variabilität zwischen den Tarifgruppen, eventuellen Zusatzverdiensten und Zuschlägen für Wochenend-/Nachtarbeit oder ähnliches, wird in der Berechnung nicht zwischen den Besoldungsgruppen unterschieden, sondern ein gemittelter Wert für die jeweiligen Dienstgruppen pro Person angenommen (Supplement Tabelle 3). Die Berechnung erfolgt minutengenau in den jeweiligen Modulen. Die Entwicklung der Personalkosten wurden berechnet anhand der jährlichen Steigerung im ÄD (6,02 %) und im PD (3,25 %).

Kosten im Modul „Schockraum“

Zusammengefasst sind die Kosten des Moduls „Schockraum“ in Tabelle 2. Der chirurgische Schockraum am UKL steht 24 Stunden an 365 Tagen im Jahr bereit.
Die Infrastrukturkosten des 47,99 m^2 großen chirurgischen Schockraums wurden in der Kostenbetrachtung über alle im Schockraum behandelten Schwerverletzten verteilt. Die Kosten setzen sich zusammen aus den Betriebskosten, Reinigung, Energie, Medizintechnik und Kaltmiete (Supplement Tabelle 4). Erhoben wurden die Daten für das Jahr 2019 und die Kosten für die Jahre 2017-2018 sowie 2020, und wurden anhand der Daten zur Sachkostenentwicklung (4,29 %) angepasst.
Die SchockraumpatientInnen werden durch den Rettungsdienst telefonisch über das „Traumahandy“ angekündigt. In einer festgelegten Telefonkaskade erfolgt die Aktivierung der jeweiligen ärztlichen und pflegerischen KollegInnen. Für diesen Arbeitsschritt vor Beginn des Schockraums werden in der Folge pauschal 7 min

veranschlagt und nach Beendigung für die ärztliche Dokumentation 10 min Arbeitszeit eines ÄD pro PatientIn eingeräumt. Für die Vorbereitung des Schockraums (Anschalten der Geräte, Vorbereiten von Infusionen und Medikamenten, Herrichten von chirurgischen Instrumenten, etc.) werden für beide PD pauschal 5 min veranschlagt, das gleiche gilt für die Nachbereitung des Schockraums (Aufräumen, Auffüllen der Verbrauchsmaterialien, pflegerische Dokumentation). Da unangekündigte Aufnahmen von PatientInnen über den Schockraum eine Seltenheit darstellen, werden diese in der weiteren Betrachtung dieser Arbeit ausgespart.

Damit kein mutmaßlich Schwerverletzter warten muss, erwartet das gesamte Schockraumteam die PatientInnen. Hierfür wurden in der Berechnung 5 min veranschlagt, was erfahrungsgemäß sehr restriktiv bemessen ist.

Der Schockraum beginnt mit der Übergabe durch den Rettungsdienst und endet mit der Verlegung der PatientInnen; dies ist die Schockraumdauer. Für die übertriagierten PatientInnen werden pauschal 30 min Schockraumdauer veranschlagt.

Das Kernteam des Schockraums setzt sich zusammen aus sieben Personen des ÄD (3xUnfallchirurgie, 2xAnästhesie/Intensivmedizin, 1xViszeralchirurgie, 1xNeurochirurgie) und zwei Personen des PD. In besonderen Fällen werden weitere ärztliche MitarbeiterInnen hinzugezogen, z.B. aus der Gynäkologie bei schwangeren Patientinnen oder Thoraxchirurgie bei schweren Verletzungen des Brustkorbs. Diese wurden nicht extra erfasst, in den Fällen von schwangeren Patientinnen oder bei Verletzungen des Brustkorbs oder Gesichts mit AIS ≥ 3 wurde ein weiterer ÄD ergänzt. Bei PatientInnen, die reanimiert werden mussten, wurden zwei weitere ÄD ergänzt.

Als übertriagiert gelten die PatientInnen, die aufgrund der Unfallanamnese zwar über den chirurgischen Schockraum aufgenommen wurden, bei denen sich aber in der Untersuchung gezeigt hat, dass sie nicht die Einschlusskriterien des TraumaRegisters DGU® erfüllen. Die bei diesen PatientInnen entstandenen Personalkosten werden auf die schwerverletzten PatientInnen umverteilt.

Für Verbrauchsgüter (Medikamente, Zugänge, Verbände, chirurgische Utensilien etc.) werden für das Jahr 2017 pauschal 100 € pro Polytrauma veranschlagt, die Kosten steigern sich jährlich entsprechend der Sachkostenentwicklung.

Kosten im Modul „OP“

Zusammengefasst sind die Kosten im Modul „OP“ in Tabelle 2. Die Kosten setzten sich zusammen aus den Kosten für die Anästhesie, den chirurgischen Personalkosten im ÄD und PD, Material- und Implantat- sowie den Infrastrukturkosten.

Die Kosten der Anästhesie wurden durch die interne Leistungsverrechnung separat erfasst. Grundlage der jährlichen Kalkulation ist die Sachkostensteigerungsrate (4,29 %).

Die Personalkosten wurden anhand der OP-Zeiten ermittelt. Angenommen wurde ein chirurgisches Team von 2,5 MitarbeiterInnen des ÄD und 2 MitarbeiterInnen des PD. Für den ÄD wurde die Schnitt-Naht-Zeit zuzüglich 30 min und für den PD die Schnitt-Naht-Zeit zuzüglich 45 min angesetzt. Grundlage der jährlichen Kalkulation sind die Personalkostensteigerungsraten für den ÄD (6,02 %) und für den PD (3,25 %).

Die Implantatkosten wurden durch die interne Leistungsverrechnung separat erfasst, die jährliche Kalkulation erfolgt anhand der Sachkostensteigerungsrate.

Zusätzlich wird für jede Operation eine Materialkostenpauschale von 50 € für Verbrauchsgüter (Handschuhe, Kittel, Kompressen etc.) und die Verwendung von vier OP-Sieben mit Kosten für die Beschaffung, Wartung, Abnutzung und Sterilisation von 250 € pauschal für das Jahr 2017 angenommen. Die jährliche Kalkulation erfolgt anhand der Sachkostensteigerungsrate.

Jeder OP-Saal unterscheidet sich leicht im Grundriss und der Grundfläche, die technische Ausstattung ist im Wesentlichen gleich. Die Infrastrukturkosten eines 50 m^2 großen Operationssaals wurden in der Kostenbetrachtung über alle behandelten Patienten der Gruppe „Gesamt“ verteilt. Die Kosten setzen sich zusammen aus den Betriebskosten, Reinigung, Energie, Medizintechnik und Kaltmiete (Berechnung entsprechend des Moduls „Schockraum“ und Supplement Tabelle 4). Es wird kein „Polytrauma-OP“ vorgehalten, sodass sich die Infrastrukturkosten nicht exakt zuordnen lassen. Hinzu kommt, dass in den Operationssälen im Regelbetrieb geplante Operationen durchgeführt werden. Zur Näherung wird davon ausgegangen, dass ein Operationssaal für die doppelte Dauer der Schnitt-Naht-Zeit (also bei 90 min: 45 min für Einschleusen, Anästhesievorbereitung, Einleiten der Narkose, Vorbereiten des Instrumentariums, Lagern und steriles Abwaschen und Abdecken des Patienten und 45 min für Verband, Ausleiten der Narkose, Ausschleusen, Aufräumen, Auffüllen und Putzen) genutzt wird. Dies wird multipliziert mit den Infrastrukturkosten eines 50 m^2 großen Operationssaals pro Minute.

Kosten im Modul „Intensivstation“

Die angefallenen Kosten auf der Intensivstation des jeweiligen Krankenhausfalls werden anhand der internen Leistungsverrechnung 2017 erfasst. Eine Anpassung der Kosten erfolgt anhand der Daten zur Sachkostenentwicklung um jährlich 4,29 %.

Kosten im Modul „Normalstation“

Für die Berechnung der angefallenen Kosten auf der Normalstation des jeweiligen Krankenhausfalls wird das Produkt aus den Behandlungstagen auf Normalstation und einem kalkulierten Tagessatz gebildet. Hierbei wird nicht zwischen Aufenthalten auf einer unfall-, viszeral- oder neurochirurgischen Stationen unterschieden. Für alle PatientInnen wird die Pflege auf einer Normalstation der Klinik für Orthopädie, Unfallchirurgie und Plastischen Chirurgie angenommen. Der Tagessatz beträgt 449,24 € für das Jahr 2017, entsprechend interner Kalkulationen. Grundlage der jährlichen Kalkulation ist die Sachkostenentwicklung (4,29 %).

Kosten im Modul „Sonstige Behandlungskosten“

Kosten für Untersuchungen und Konsilleistungen

Die Einzelkosten werden in der internen Leistungsverrechung des UKL für jeden Behandlungsfall einzeln erfasst: Radiologische und nuklearmedizinische Diagnostik und Interventionen (inklusive Materialkosten), laborchemische Untersuchungen (ohne Transfusionsmedizin) und Untersuchungen der Institute für Pathologie, Mikrobiologie, Virologie und Immunologie sowie konsiliarärztliche Mitbehandlung durch andere Fachabteilungen. Die Anpassung der Konsil-Kosten erfolgt anhand der Personalkostenentwicklung im ÄD um jährlich 6,02 %. Die Anpassung der restlichen Kosten erfolgt anhand der Sachkostenentwicklung um jährlich 4,29 %.

Kosten für Transfusionsmedizin

Die Einzelkosten der jeweiligen Untersuchungen und der Bereitstellung von Transfusionsprodukten werden in der internen Leistungsverrechnung des UKL für jeden Krankenhausfall einzeln erfasst. Die Menge der verabreichten Transfusionsprodukte in der Schockraumphase und im gesamten Krankenhausaufenthalt wurden fallbezogen dokumentiert. Zusätzliche Kosten (z.B. Bedside-Test und Infusionsbestecke) sind als pauschale Verbrauchsgüter in den Modulen „Schockraum“ und „OP“, sowie in der internen Leistungsverrechnung der

Intensivstation und dem Tagessatz der Normalstation erfasst. Die Anpassung der Kosten erfolgt anhand der Sachkostenentwicklung um jährlich 4,29 %.

Kosten für Ergotherapie, Logopädie und Physiotherapie

Die angefallenen Kosten für Ergotherapie, Logopädie und Physiotherapie des jeweiligen Krankenhausfalls werden anhand der internen Leistungsverrechnung 2017 erfasst. Die Anpassung der Kosten erfolgt anhand der Daten zur Personalkosten-entwicklung im PD um jährlich 3,25 %.

Kosten im Modul „Overhead"

Netzwerk-Kosten durch Organisation

Die zeitliche Belastung des diensthabenden unfallchirurgischen OberarztIn (Traumaleader) des UKL durch Verlegungsanfragen im Traumanetzwerk Westsachsen wurde beispielhaft für den Monat Juli 2019 erfasst. Hierbei wurde unterschieden zwischen Konsilanfragen und Verlegungsanfragen. Dokumentiert wurden alle eingehenden Telefonate an das Traumahandy, der Grund des Anrufs und die Gesprächsdauer. Betrachtet wurden nur die Verlegungsanfragen. Die Arbeitszeitkosten werden entsprechend der Personalkostenentwicklung im ÄD kalkuliert.

Kosten für Mehraufwand durch Dokumentation und Zertifizierung

Durch die Beteiligung des UKL am TraumaRegister DGU® und am Traumnetzwerk Westsachsen entstehen zusätzliche Kosten für u.a. Dokumentation und Zertifizierung. Es erfolgte eine Kostenaufstellung für die Jahre 2014-2018 im Rahmen der klinikinternen Budgetplanung (bespielhaft gezeigt für das Jahr 2017 in Supplement Tabelle 5). Für die Folgejahre 2019-2020 wurde ein gleichbleibender Kostenanstieg entsprechend der Entwicklung der fünf vorherigen Jahre angenommen (4,88 %). Nicht eingerechnet sind klinikgetragene Kosten für notwendige Weiterbildungen des ÄD und PD (z.B. ATLS®-Kurse). Ebenfalls nicht berücksichtigt sind die UKL-spezifischen Kosten des Arbeitsaufwands (der Autoren CJ und JF) durch die Sprecherfunktion im Traumanetzwerk Westsachsen.

Nicht erfasste Kosten

Bautechnische Kosten und Anschaffungskosten für medizinische Geräte

Unter der dualen Finanzierung in der stationären Versorgung entfällt dieser Punkt aus Sicht des Krankenhauses und ist deswegen nicht Teil der Betrachtung.

Kosten im prä- und poststationären Bereich

Die Kosten für die präklinische Rettung sowie die Kosten für die Anschlussheilbehandlung oder Rehabilitation entfallen aus Sicht des Krankenhauses und wurden nicht berücksichtigt.

Indirekte oder intangible Kosten

Nicht berücksichtigt wurden Kosten, die durch Ausfall des Arbeitskraftpotentials entstanden sind. Ebenfalls nicht berücksichtigt werden Kosten, aus unfallresultierenden Einschränkungen wie Schmerz, Funktionsverlust, Depressionen oder allgemeinem Verlust an Lebensqualität.

Kostenschätzer im TraumaRegister DGU®

Lefering et al. beschrieben eine Möglichkeit, durch Addition von 7 Multiplikatoren die Kosten in der Schwerverletztenversorung zu überschlagen [4]. Die Validierung des Kostenschätzers erfolgte für das Jahr 2007/2008, die Anpassung der Einzelkosten erfolgt anhand der Daten zur Fallkostenentwicklung um jährlich 2,93 % (Zusatzmaterial online Tabelle 8).

II. Supplement Ergebnisse - Modul „Sonstige Behandlungskosten“

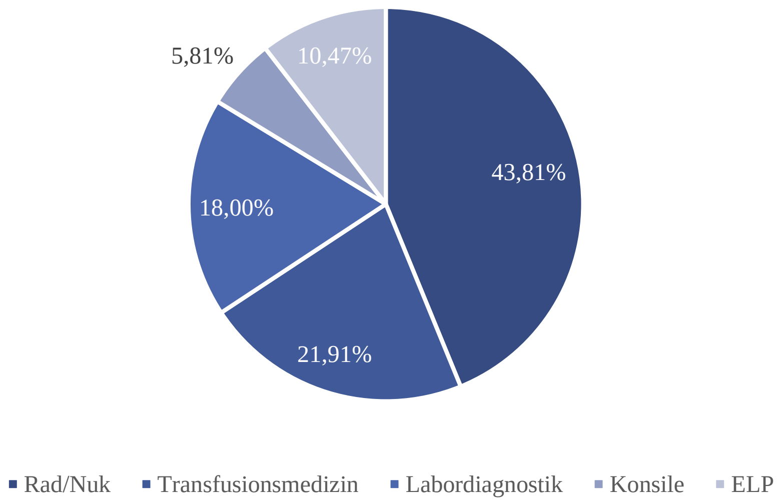

Supplement Fig. 1 – Kostenverteilung im Modul „Sonstige Behandlungskosten“ im Jahr 2017. Rad/Nuk – Klinik für diagnostische und interventionelle Radiologie und Klinik für Nuklearmedizin, ELP – Ergotherapie, Logopädie und Physiotherapie.

III. Supplement Literaturverzeichnis

1. Statistisches Bundesamt. Sachkosten deutscher Krankenhäuser insgesamt in den Jahren 2000 bis 2017 (in Milliarden Euro) [Internet]. 2018. Available from: https://de.statista.com/statistik/daten/studie/169301/umfrage/sachkosten-deutscher-krankenhaeuser-insgesamt-seit-2000/
2. Statistisches Bundesamt. Personalkosten deutscher Krankenhäuser im Pflege- und ärztlichem Dienst in den Jahren 2000 bis 2017 (in Millionen Euro) [Internet]. Statista GmbH; 2018. Available from: https://de.statista.com/statistik/daten/studie/169305/umfrage/personalkosten-deutscher-krankenhaeuser-pflege--und-aerztlicher-dienst-seit-2000/
3. Statistisches Bundesamt. Bereinigte Kosten je Behandlungsfall in deutschen Krankenhäusern in den Jahren 1991 bis 2017 (in Euro je Behandlungsfall). [Internet]. Statista GmbH; 2018. Available from: https://de.statista.com/statistik/daten/studie/322429/umfrage/bereinigte-kosten-je-behandlungsfall-in-deutschen-krankenhaeusern/
4. Lefering R, Mahlke L, Franz D. Der Kostenschätzer im TraumaRegister DGU®. Unfallchirurg [Internet]. 2017;120(12):1065–70. Available from: https://doi.org/10.1007/s00113-016-0266-1
